# Effect of honey solution and water acquisition on survival of starved solenopsis mealybug, *Phenacoccus solenopsis*

**DOI:** 10.1093/jis/14.1.1

**Published:** 2014-01-01

**Authors:** Fang Huang, Feifei Wang, Yaobin Lu, Pengjun Zhang, Jinming Zhang, Zhijun Zhang, Weidi Li, Wencai Lin, Yawei Bei

**Affiliations:** State Key Laboratory Breeding Base for Zhejiang Sustainable Pest and Disease Control, Institute of Plant Protection and Microbiology, Zhejiang Academy of Agricultural Sciences, Hangzhou, Zhejiang 310021, China

**Keywords:** artificial membrane, cotton mealybug, electrical penetration graph technique, invasive pest, starvation

## Abstract

The current study examined the effects of honey solution and water access on feeding behavior and survival of starving solenopsis mealybugs, *Phenacoccus solenopsis* Tinsley (Hemiptera: Pseudococcidae). The electrical penetration graph technique and an artificial membrane system were used to check whether *P. solenopsis* could imbibe free water or other liquid, such as the honey solution used here, in its natural environment. The recorded electrical penetration graph waveforms revealed that *P. solenopsis* could continuously imbibe water-honey solution for several hours, which indicated that honey solution and water acquisition could possibly occur when *P. solenopsis* had access to such liquids in its natural environment. Waveforms of water-honey solution feeding alternated between two distinct feeding phases in a regular pattern, which was assumed to reflect inherent habits of feeding attempts. The effects of honey solution and water acquisition on survival of *P. solenopsis* was also examined. Comparison between *P. solenopsis* in different treatments (starved, water feeding, honey solution feeding, and cotton plant feeding) suggested that 1) *P. solenopsis* could accept but did not favor feeding on water or the honey solution, and 2) this feeding could prolong its survival, but had no effect on body size.

## Introduction

The solenopsis mealybug, *Phenacoccus solenopsis* Tinsley (Hemiptera: Pseudococcidae), is a sap-sucking pest of many agricultural, horticultural, and ornamental crops, including cotton ( Dutt 2007 ; Wang et al. 2010 ). Recently, it has caused serious economic losses in transgenic and conventional cotton production in India ( Jhala et al. 2008 ) and Pakistan ( Abbas et al. 2009 ). Its polyphagous nature, high reproductive capacity, and short life cycle ( Huang et al. 2011 ), and wide morphological plasticity have enabled *P. solenopsis* to survive and even thrive under a wide variety of environmental conditions ( Hodgson et al. 2008 ). The species has spread rapidly in Asia and beyond, threatening the world’s cotton industry and other crops as an invasive pest ( Wang et al. 2010 ). Consequently, *P. solenopsis* has been added to the European and Mediterranean Plant Protection Organization list ( EPPO 2011 ) and Chinese quarantine pest list ( MOA and AQSIQ 2009 ).

Population fitness homoeostasis, defined as the ability of an individual or population to maintain relatively constant fitness over a range of environments, determines species invasiveness in large part ( Rejmánek 2000 ). Within parameters of this ‘fitness,’ resistance to food deprivation was suggested to be an important adaptation in flightless insects ( Stockhoff 1991 ). Under severe defoliation, individuals may be forced to wander extensively to locate other hosts. Utilization of liquid (dew and/or proximal honey solution) in the environment was reported to be important for survival of some insects, i.e., *Drosophila* ( Karan et al. 1998 ), mites ( van Rijn and Tanigoshi 1999 ), mosquitoes ( Gary and Foster 2004 ), and many coccinellids ( Lundgren 2009 ). Little information could be found to reveal whether or how plant sap-sucking insects with specialized mouthparts could use these fluids. Mittler and Dadd (1963) used the electronic penetration graph technique (EPG) and confirmed that the green peach aphid, *Myzus persicae* , could ingest considerable amounts of water and sugary fluids entirely by their own sucking efforts. Kehat and Wyndham (1972) reported that survival of the female Rutherglen bug, *Nysius vinitor* , with access to water was uninfluenced. If starving, could *P. solenopsis* consume water and/or other fluids when offered, and will this consumption increase its odds of survival? It has been suggested that *P. solenopsis* , as an invasive pest with limited mobility, was dispersed mainly through commercial transportation ( Giliomee 2011 ; Fand 2013 ). Travel between suitable locations is potentially a lengthy process for *P. solenopsis* , and changes in food quality, falling from the host plant, and food depletion could occur during movement. If dew or another fluid could be used by *P. solenopsis* , individuals would be expected to survive longer, which would improve their chances to find a suitable environment. In other words, benefits from the utilization of these fluids could promote their survival in a new environment, which is an essential trait for an invasive insect.

The EPG technique was originally derived from the electronic feeding monitor, and it allows realtime tracking of the invisible stylet penetration (probing) activities by insects with piercing-sucking mouthparts ( Tjallingii and Hogen Esch 1993 ; Walker 2000 ). An artificial membrane system that made the process of water sucking visible was developed ( Huang et al. 2013 ) in conjunction with EPG. Plant sap sucking procedures of *P. solenopsis* were described, and water being sucked through a stretched parafilm membrane was observed in a previous study ( Huang et al. 2012 ).

To confirm whether water consumption is beneficial, the present study was conducted to investigate whether access to honey solution or water influenced the feeding behavior and survival of starving *P. solenopsis* . The importance of the consumption of these substances to the invasive ability of the species is discussed.

## Materials and Methods

### Insects and plants

*Phenacoccus solenopsis* specimens were originally collected from the ornamental plant Rose of Sharon, *Hibiscus syriacus* L. (Malvales, Malvaceae), in Hangzhou, China. The specimens were reared on cotton, *Gossypium hirsutum* L., cultivar Zhefengmian No.1, in a climate-controlled room at 27 ± 1º C, RH 65~75%, and 12:12 L:D. The *G. hirsutum* plants for use in the culture were grown in 13 cm plastic pots in a mixture of peat moss, *Sphagnum* sp. (Sphagnalus, Sphagnaceae), vermiculite, organic fertilizer, and perlite (10:10:10:1 ratio), and kept in a climate-controlled room (25 ± 3º C, R.H. 60~ 70%, 12:12 L:D). All the plants used in the experiments had two or three fully-expanded leaves.

### EPG analysis

The stylet penetration activities of *P. solenopsis* on the artificial membrane were recorded by a Giga-4 DC-EPG system (EPG Systems, http://epgsystems.com ). Newly-emerged *P. solenopsis* adults were carefully collected from the colony and starved for 24 hr. Their dorsal wax was partially removed with a long-haired goat’s wool brush to permit attachment of an EPG insect electrode (a thin gold wire, diameter 18 µm, length 2 cm) using a droplet of water-based silver glue. The insect electrode (+ve) was connected to the EPG ampli-amplifier, which had an input resistance of 10 ^9^ ohm and a gain of 50×( Tjallingii 1985 ). In assessments of water or honey solution acquisition, the other electrode (-ve) was inserted into the liquid through the Parafilm (BEMIS, www.parafilm.com ) membrane. Assessment of EPG on the *G. hirsutum* plant was set up as a control and was conducted using the same materials and methods as Huang et al. (2012) . Test material was placed in a Faraday cage at an ambient temperature of 27 ± 2° C to shield it from external electric noise. All recorded signals were analyzed using Probe 3.4 software (provided by W.F. Tjallingii, Wageningen, The Netherlands).

Before bioassay, *P. solenopsis* was held above the Parafilm using an attached gold wire and then lowered onto the membrane surface, at which point EPG output was recorded. Preliminary overview of stylet activity was observed continuously for a 24-hr period. Data of 12-hr recordings were used to analyze EPG variables. In each treatment, EPG signals of 80 individual *P. solenopsis* specimens were recorded, and no less than 20 sets of EPG data were analyzed.

### Female adults’ survival and body size

To evaluate the effect of water or honey solution acquisition on *P. solenopsis* survival rates, body size and survival time were examined for each specimen.

Three hundred newly-emerged adult female *P. solenopsis* were isolated individually in 9 cm Petri dishes prior to being randomly allocated to one of the following four treatments: (1) access to water (WM) (n = 80): a Petri dish containing a *P. solenopsis* specimen was covered with a 5 µm thick membrane and then upended over distilled water, which was changed daily; (2) access to honey solution (NM) (n = 80): a Petri dish containing a *P. solenopsis* specimen was set up using the same procedure as the WM treatment, except that they were upended over honey solution, which was changed daily; (3) starvation (SV) (n = 60): females were kept individually in empty Petri dishes covered by Parafilm; (4) control (CRL) (n = 80): each Petri dish base was lined with a piece of moist filter paper, on which *G. hirsutum* leaves were placed adaxi-ally; the leaves were replaced daily. Each *P. solenopsis* specimen was teased with a fine brush into a non-feeding mode without injury, then carefully transferred onto the new leaves by a long-haired goat’s wool brush. Each Petri dish was then covered by a 5 µm thick membrane. *P. solenopsis* in Petri dishes were checked twice daily (at 08:00 and 20:00) to record their survival rates. When the insects died, their body size (length and maximum width) was measured.

### Statistics

Body size and EPG data were analyzed using analysis of variance (ANOVA) followed by Tukey’s HSD test.

## Results

### EPG waveforms of *P. solenopsis* in water and honey solution conditions

**Figure 1.**
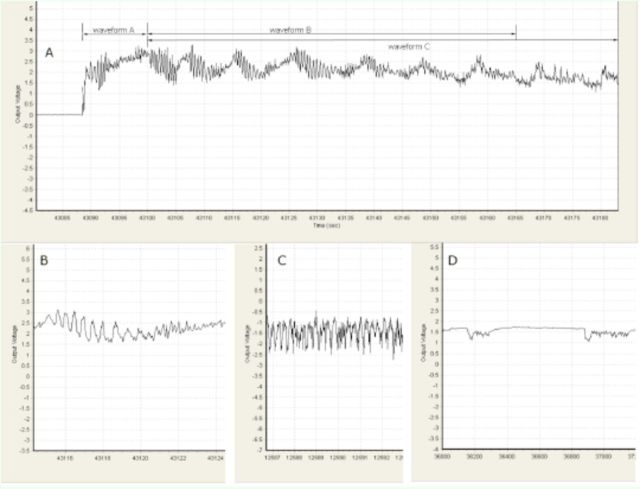
EPG waveforms recorded from *Phenacoccus solenopsis* in the WM treatment (artificial membrane on distilled water). A: waveforms of the phase I feeding pathway, including waveforms A, B and C; B: waveform C; C: waveform of water acquisition (waveform G); D: alternate feeding in phase II. High quality figures are available online.

In the WM treatment, the four major and distinct EPG waveforms recorded were characterized and labeled as A, B, C, and G ( Figure 1 ). Waveform A occurred at the commencement of each probe; it showed the greatest amplitude, with high frequency and irregular waveforms ( Figure 1A ). Waveform B, which followed waveform A, was characterized by gradually decreasing amplitude over time and usually had fewer than 10 repetitions ( Figure 1A ). Waveform C coincided with or was superimposed on waveform B ( Figure 1A ). When waveform B faded away, waveform C remained with a continuation of the B wave periodicity, as in most of the signal shown in Figure 1B . Waveform G ( Figure 1C ) was the only waveform directly related to feeding activity and was characterized by a frequency of 3–6 Hz.

**Figure 2.**
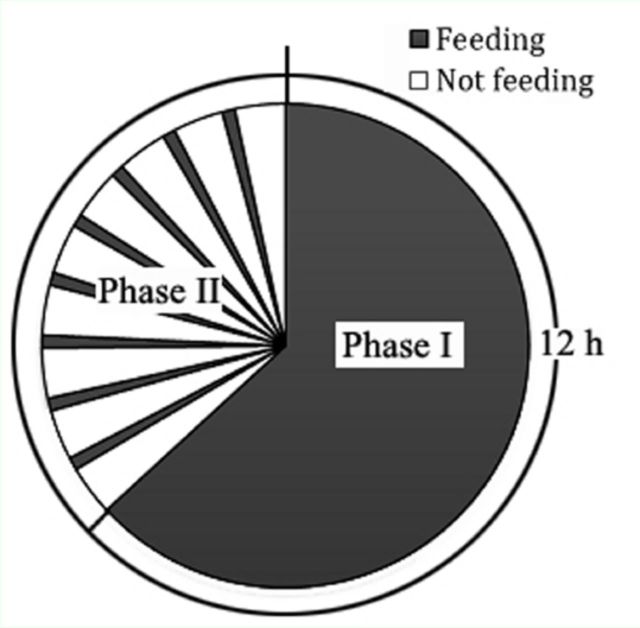
EPG signals of *Phenacoccus solenopsis* feeding on water on artificial membrane, showing incidence of phases I and II in repeated 12-hr period. High quality figures are available online.

Observations of EPG waveforms recorded from *P. solenopsis* on artificial membranes revealed repetition in some sort of pattern at 12 hr intervals ( Figure 2 ). During each 12 hr period, two distinct phases could be determined: a continuous feeding phase (phase I) and an alternating feeding phase (phase II, Figure 2 ). In phase I ( Figures 1A , B , and C ), *P. solenopsis* were generally feeding continuously, but some non-probing events were observed for a few individuals. In phase II, periods of non-feeding without new probing activities (~ 10 min) regularly alternated with periods of feeding (2–7 min) ( Figure 1D ).

**Figure 3.**
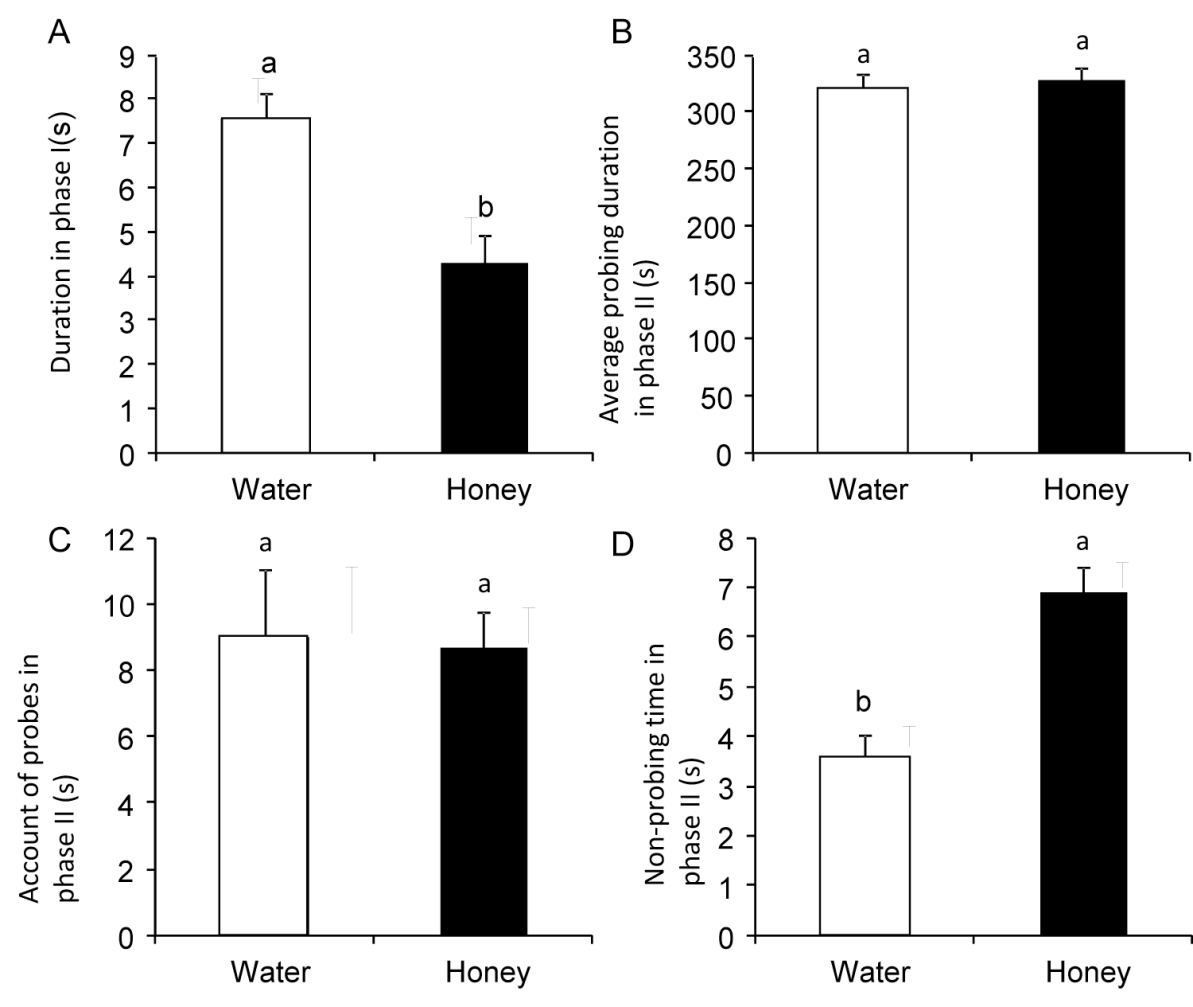
Characteristics of EPG waveforms of *Phenacoccus solenopsis* fed on water-honey solution through artificial membranes over 12 hr. Different letters above the bars indicate significant difference at *p* < 0.05, as determined by oneway ANOVA. High quality figures are available online.

In the NM treatment, although the same waveforms as above were recorded, some differences between the treatments were nevertheless present in the parameters of phases I and II ( Figure 3 ). Compared to the WM data, in NM the duration of phase I was significantly shorter. The average duration of probing and the number of feedings in phase II were not significantly different between the treatments, but the duration of non-probing time was significantly longer in NM than in WM.

### Comparison of *P. solenopsis* feeding behavior among different treatments

Due to the simple EPG waveforms recorded on the artificial membrane, analysis of *P. solenopsis* EPG waveforms in the previous report ( Huang et al. 2012 ) was simplified as follows: (1) potential drop waveform was pooled into waveform C, and (2) data of phloem feeding-related waveforms were used to describe the sap-sucking activities ( Huang et al. 2012 ), which were represented by waveform G in WM and NM treatments. The results of the comparison between the two treatments and the CRL are shown in Table 1 . *P. solenopsis* initiated probing into plant leaf tissue in 6 min, whereas it took at least 1 hr to occur in the NM treatment and even longer in the WM treatment. *Phenacoccus solenopsis* feeding on artificial membranes probed more often than when on plant tissue. *Phenacoccus solenopsis* on *G. hirsutum* leaves spent the longest time probing, followed by *P. solenopsis* in the WM treatment. In the feeding phases, *P. solenopsis* in NM spent the least time feeding; total feeding time was longest in WM. *Phenacoccus solenopsis* in NM spent the most time in non-probing activity, followed by WM; insects in the CRL spent the least time in non-probing activity (< 1.5 hr in a 24-hr period). The percentage of feeding time spent probing was calculated based on the above data. The time spent probing *G. hirsutum* leaves in the CRL (32.20 ± 4.65%) was much shorter than in the other treatments (86.67 ± 2.91% and 84.30 ± 3.02% in WM and NM, respectively).

**Table 1.**
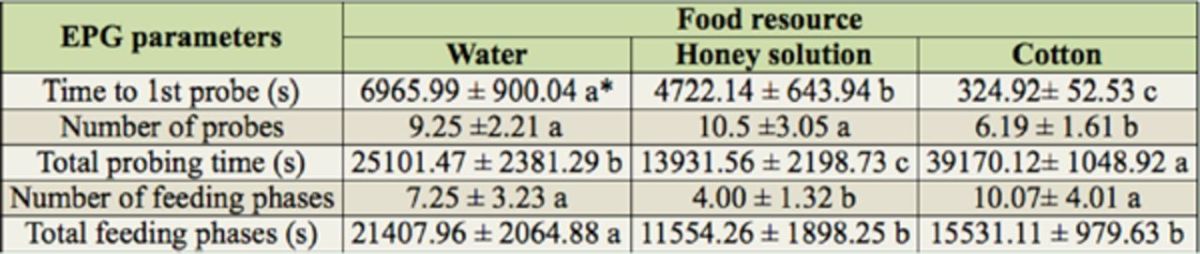
Characteristics of EPG waveforms of *Phenacoccus solenopsis* feeding through artificial membranes and on cotton leaves.

Data in the same row followed by a different letter indicate significant difference ( *p* < 0.05) among treatments, determined by ANOVA followed by Tukey’s HSD test. ǂTotal time is 12 hr.

### Body sizes of *P. solenopsis* in different treatments

**Figure 4.**
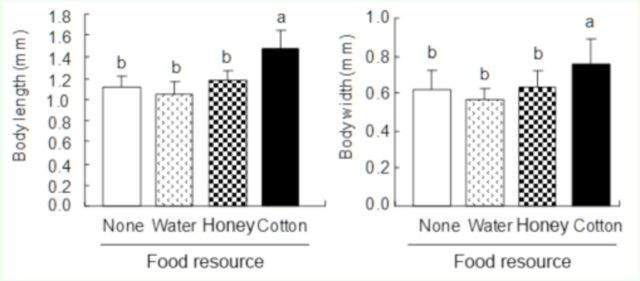
Body sizes of dying *Phenacoccus solenopsis* in different food source treatments (n = 80 per treatment). Different letters above the bars indicate significant difference at *p* < 0.05, as determined by ANOVA followed by Tukey’s HSD test. High quality figures are available online.

The effect of different food source treatments on *P. solenopsis* body size was significant (length: *F* 3,51 = 32.0, *p* < 0.001; width: *F* 3,51 = 7.8, *p* < 0.001). Measured upon death, *P. solenopsis* in the SV, WM, and NM treatments were significantly smaller than those in the CRL ( Figure 4 ). Body dimensions were 1–1.2 mm long and 0.5–0.7 mm wide in the three former treatments, compared with > 1.4 mm long and > 0.7 mm wide in the CRL treatment.

### Survival of adult *P. solenopsis* in different treatments

**Figure 5.**
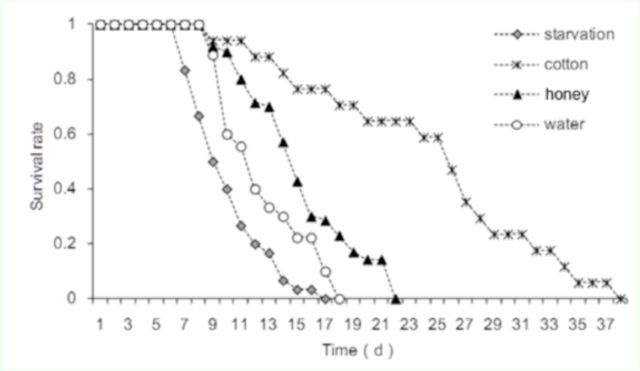
Survival curves for adult *Phenacoccus solenopsis* in different treatments from the time of emergence. High quality figures are available online.

**Figure 6.**
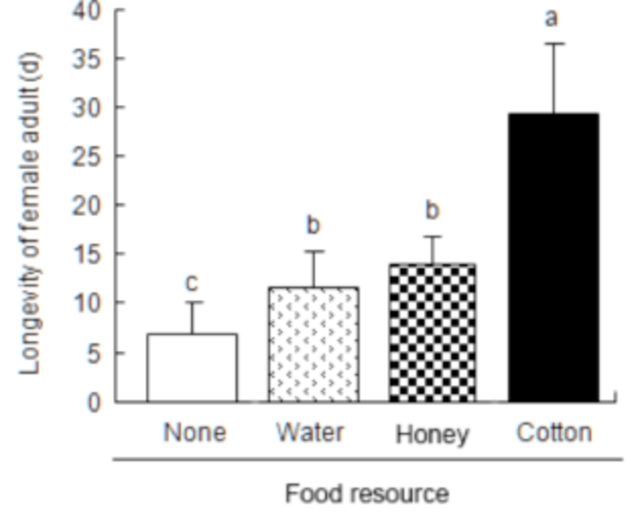
Longevity of adult female *Phenacoccus solenopsis* in different treatments. Different letters above bars indicate significant difference at *p* < 0.05 as determined by ANOVA followed by Tukey’s HSD test. High quality figures are available online.

Different feeding treatments significantly influenced survival of newly-emerged adult female *P. solenopsis* . Some individuals in the starved group began to die within five days, whereas adults in other groups survived at least one week ( Figure 5 ). Survivorship was progressively higher for *P. solenopsis* in WM, NM, and CRL treatments ( Figure 6 ). The effect of food treatments on longevity was significant ( *F* 3,50 = 85.1, *p* < 0.001). The average longevity of *P. solenopsis* was longest (29.26 ± 7.42 days) in the *G. hirsutum* leaf treatment compared to the other three treatments. The adults that fed on water and honey solution survived for 11.67 ± 3.5 days and 14.17 ± 2.79 days, respectively (no significant difference). Starved mealybug adults only lived for 6.83 ± 3.13 days.

## Discussion

Compared to the structure of *G. hirsutum* leaf tissue, the artificial membrane system that *P. solenopsis* penetrated to get water was comparatively simple, so only two types of EPG waveforms were recorded, i.e., waveforms for penetration and water ingestion. These waveforms occurred alternately in two distinct feeding phases, a phase of continuous feeding and a phase of alternation between penetration and feeding. The continuous feeding phase could last for several hours, indicating that *P. solenopsis* could imbibe water. In previous work, EPG waveforms of *P. solenopsis* on *G. hirsutum* involving signals of ingestion from xylem tissue were reported ( Huang et al. 2012 ). Such xylem ingestion rarely occurred on *G. hirsutum* , but it could last for a considerable period of time once it commenced. This suggests that the *P. solenopsis* might ingest water, which was confirmed in the current study, in which a treatment with a supply of honey solution was set up to make a parallel comparison. The results showed that the feeding behavior characteristics of *P. solenopsis* on an artificial membrane with different liquids underneath were similar to some extent, showing the same pattern. Such a regular pattern did not occur when *P. solenopsis* were feeding on *G. hirsutum* , which might be an inherent habit for feeding attempts when *P. solenopsis* encounter only unsuitable hosts. Such a hypothesis has yet to be confirmed.

*Phenacoccus solenopsis* individuals consumed water and a honey solution when these were made available. Their feeding behavior on these liquids was examined and compared to that on *G. hirsutum* . EPG data comparison of *P. solenopsis* in different treatments revealed that *P. solenopsis* on *G. hirsutum* leaves began to penetrate the tissues within 10 min, whereas *P. solenopsis* on the artificial membrane did not start to probe until > 1 hr had passed. In addition, on *G. hirsutum* leaves ~ 90% of the recording time was associated with stylet activities, while only 58% and 32% of the time was spent on these activities on the artificial membranes over water and honey, respectively. Moreover, stylet evulsion during probing in cotton plant tissues occurred once per two hours, whereas such probing frequency was doubled on the artificial membranes. These results suggest that *P. solenopsis* ac-cepts but does not prefer feeding on water. The results in this study were obtained using the EPG system with an artificial membrane. Water in the natural environment would be devoid of a surrounding membrane; thus, whether *P. solenopsis* would directly imbibe free water still needs to be confirmed.

The effect of feeding on water in terms of *P. solenopsis* survival was also studied. The longevity in treatments was SV < WM/NM < CRL, suggesting that imbibing water can prolong *P. solenopsis* survival. However, feeding on water alone did not significantly increase *P. solenopsis* body size compared to feeding on *G. hirsutum* leaves. Feeding on honey solution may have prolonged *P. solenopsis* survival, but it did not increase their body size.

In conclusion, the results of the current study suggest that water-honey solution acquisition through an artificial membrane may be a result of inherent feeding habits and may be of survival benefit to *P. solenopsis* . This assumed inherent feeding behavior suggests that *P. solenopsis* might probe into any plant it contacts. If the content of plant phloem is ideal for *P. solenopsis* nutrition, the water in the phloem and xylem may also be of survival benefit. *Phenacoccus solenopsis* has been recorded on plants belonging to more than 100 genera in over 50 families ( Abbas et al. 2009 ), but the suitability of many of these host plants has yet to be determined. If *P. solenopsis* will probe any plant, the suitability of different plant hosts must be examined to distinguish which hosts are permanently suitable and which serve as temporary hosts. Such information would be valuable for integrated pest management of *P. solenopsis* .

## Abbreviations

CRL: control treatment
EPG: electrical penetration graph
NM: access to honey solution treatment
SV: starvation treatment
WM: access to water treatment
